# Laparoscopic Nephroureterectomy for Adult Patient with Primary Obstructive Megaureter

**DOI:** 10.1155/2013/124710

**Published:** 2013-12-22

**Authors:** Kimito Osaka, Kazuhide Makiyama, Shinji Ohtake, Hiroyuki Yamanaka, Futoshi Sano, Noboru Nakaigawa, Yoshinobu Kubota

**Affiliations:** Department of Urology, Yokohama City University Graduate School of Medicine, 3-9 Fukuura, Kanazawa-Ku, Yokohama 236-0004, Japan

## Abstract

A 29-year-old female with a complaint of abdominal distension was referred to our hospital. She had a history of being treated for pyelonephritis three times. By computed tomography and retrograde pyelography, she was diagnosed with adult left primary megaureter. Her left renal function was severely deteriorated. She hoped for surgical intervention before becoming pregnant. Laparoscopic nephroureterectomy for megaureters seems to be difficult due to the large size. By sucking urine from an inserted ureteral catheter and setting trocar positions, we successfully performed laparoscopic nephroureterectomy for megaureter.

## 1. Introduction

Primary obstructive megaureter (POM) is a common disease in children, although it is uncommon in adults. The condition is characterized by an intrinsic congenital obstruction at the lower end of the ureter [1]. Today, many cases are detected in utero by antenatal ultrasonography [2]. POM in children is usually asymptomatic and can be treated conservatively in most cases [3]. However, compared with the clinical course in children, POM in adults is different.

POM in adults can present with flank pain, recurrent urinary tract infection, and urolithiasis. In addition to these complications, little spontaneous regression can be anticipated by the maturation of the vesicoureteral junction [4]. POM in adults requires more aggressive surgical interventions than POM in childhood. We present a case of laparoscopic nephroureterectomy for a young adult female with POM.

## 2. Case Presentation

**Figure 1:**
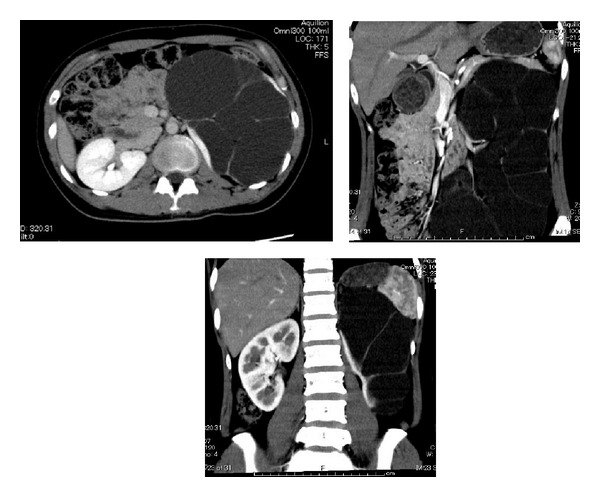
Computed tomography shows dilated left urinary tract and thin renal parenchyma.

**Figure 2:**
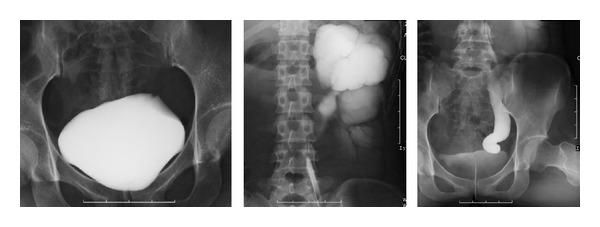
Cystography shows absence of vesicoureteric reflux and retrograde pyelography shows dilated left renal pelvis and full length of ureter.

**Figure 3:**
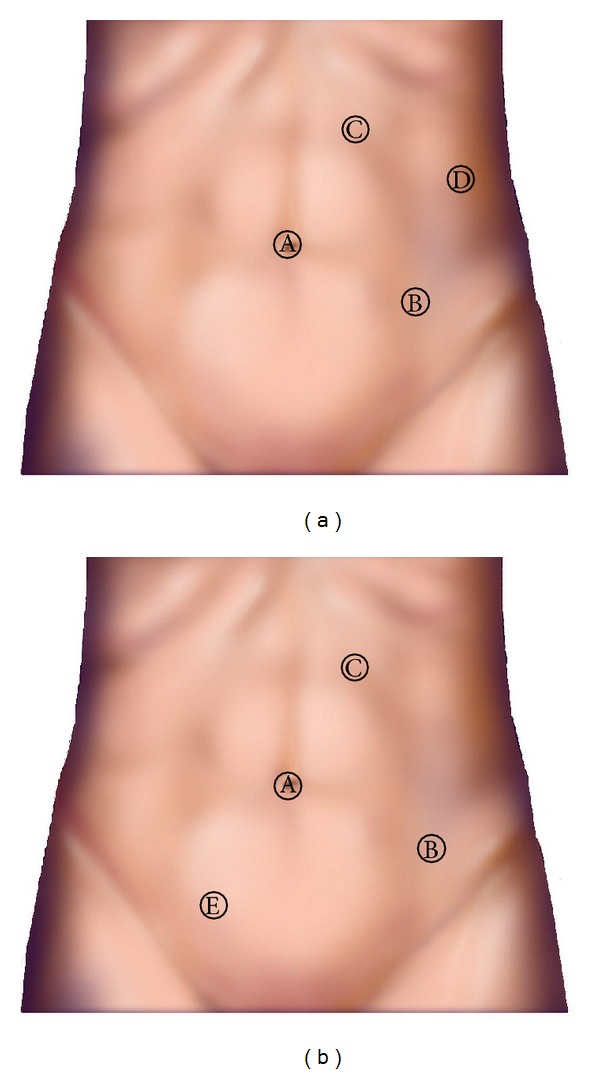
Port placement. (a) Right flank position. (b) Head-down position.

A 29-year-old female with a complaint of abdominal distention was referred to our hospital. She had been treated with antibiotics for acute pyelonephritis three times when she was 6, 20, and 28 years old. Blood test results including renal function and urine test were normal. Abdominal computed tomography (CT) scan demonstrated markedly dilated left calyceal structures, a pelvis with thinning renal parenchyma, and left ureter from the proximal to the distal end (Figure 1). Retrograde pyelography revealed the absence of anatomical malformation of the ureteral orifice and the absence of vesicoureteral reflux (Figure 2). Urinary cytology of the left ureter was negative. Split function of the left kidney function was less than 10% in renal scintigraphy. The diagnosis was megaureter due to stricture of the vesicoureteral junction. The patient hoped for surgery before becoming pregnant. Laparoscopic nephroureterectomy was performed by an intraperitoneal approach. Prior to the laparoscopic procedure, an open-ended 6Fr left ureteral stent was placed. During surgery, the patient was initially positioned in the right flank position and four trocars were placed. The pneumoperitoneum pressure was 10 mmHg. The first 12 mm trocar (A: camera port) was inserted in the umbilicus. The second 12 mm trocar (B) was located in the anterior axillary line above the ilial crest. The third 5 mm trocar (C) was located in the midclavicular line on the left. The fourth 5 mm trocar (D) was placed on the upper lateral site of the third trocar for assisting instruments (Figure 3).

We approached the retroperitoneum from the lateral aspect of the descending colon. To facilitate dissection around the kidney, the ureter was emptied by sucking urine through a placed ureteral stent. After circumferential dissection of the kidney, we cut the renal artery and vein with polymer ligating clips. Then, the patient was positioned in a Trendelenburg position and a 5 mm trocar was inserted in the right lower umbilicus (E). The surgeon changed his right-hand trocar from (B) to (E) and his left-hand trocar from (C) to (B). The sigmoid colon was reflected medially and the dilated ureter was dissected down to the bladder. Then, the ureteral stent was removed and we resected the distal end of the ureter with polymer ligating clips. We extracted the resected specimen from the first trocar extending the skin incision to 30 mm using a 15 mm endocatch bag. The specimen weighed 576 g.

The total operative time was 262 minutes. Circumferential dissection of the left kidney in the right flank position took 129 minutes and resection of the distal ureter and removal of the specimen in a head-down position took 83 minutes. The total pneumoperitoneum time was 212 minutes. The estimated blood loss was minimal. The urine volume sucked from the inserted ureteral catheter was 900 mL.

**Figure 4:**
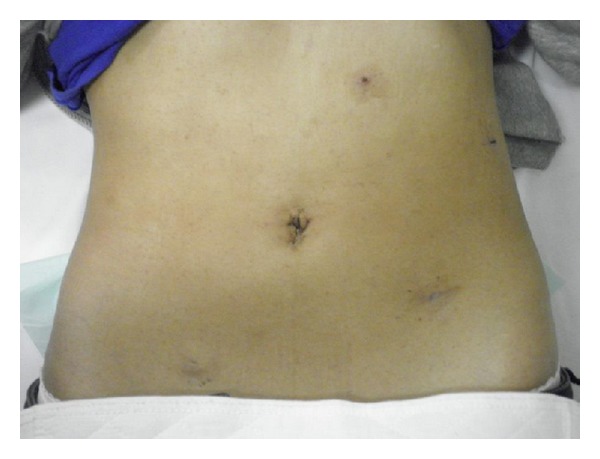
Postoperative wound.

Operative and pathological findings showed that the kidney size was 12 × 8 × 6 cm, renal pelvis was remarkably dilated, and, in some parts of the renal parenchyma, interstitial fibrosis was recognized. The patient was discharged from our department without complications and was satisfied with her postoperative wound (Figure 4).

## 3. Discussion

POM due to congenital anomaly of the lower end of the ureter is on rare occasions identified in adults. The disease typically presents in the third or fourth decade of life. Males are more commonly affected than females [5]. Unilateral disease is more common and is usually on the left side [6]. The disease can present flank pain, hematuria, recurrent urinary tract infection, urolithiasis, and reduced renal function [5]. The management in children is conservative in many cases because spontaneous resolution of the dilation is often seen on regular followup [3]. In contrast to the presentation in children, most cases of POM in adults are symptomatic.

In view of the variable symptoms, active surgical interventions are usually required [4]. Most patients undergo excision of the narrow segment and ureteroneocystostomy with tapering of the dilated ureter, and some patients whose renal function is impaired or severely deteriorated undergo nephroureterectomy [4]. In cases presenting urolithiasis, most of the urolithiasis can be removed at the time of ureteroneocystostomy, or by endoscopic methods or by extracorporeal shock wave lithotripsy [4].

In our case, the patient had recurrent pyelonephritis and discomfort of distended abdomen.

She hoped for surgery before marriage and pregnancy. CT scans showed that the thickness of the left renal parenchyma was less than 1 cm and split function of the left renal kidney was less than 10%. Her left renal function was severely deteriorated, so we selected left nephroureterectomy. We chose a laparoscopic approach to reduce the morbidity and improve cosmetic aspects and recovery time.

Recently, there have been some reports of laparoscopic and robotic ureteroneocystostomy of the megaureter [7, 8]. However, open nephroureterectomy currently remains the standard surgery for megaureters because laparoscopic nephroureterectomy for megaureters seems to be difficult. Laparoscopic nephroureterectomy for megaureters requires extensive dissection of the dilated ureter and enlarged kidney, as well as a wide intraperitoneal working space.

In our case, we could reduce the size of the kidney and ureter by sucking urine from a previously inserted ureteral catheter. This procedure facilitated the dissection of the kidney. After dissection of the kidney and resection of the renal vessels, the patient was placed in the Trendelenburg position from the right flank position. In addition to changing the patient's position, we added a 5 mm trocar (E) in the lower right of the umbilicus for the surgeon's right hand and changed his left-hand trocar from (C) to (B). By changing the position and trocars, we made a wide intra-abdominal working space and could perform laparoscopic nephroureterectomy. This patient was a young female, who was satisfied with her postoperative wound. Laparoscopic nephroureterectomy may be a viable treatment method for POM of nonfunctional and severely deteriorated function of the kidney.
